# Supplementary material for: Cloud BioLinux: pre-configured and on-demand bioinformatics computing for the genomics community
Source: BMC Bioinformatics. 2012 Mar 19;13:42. doi: 10.1186/1471-2105-13-42 (PMC3372431; doi:10.1186/1471-2105-13-42)
Supplement: Additional file 1 — Supplementary 1 Cloud BioLinux software documentation in the form of a mini, self-contained website. Users need to download and uncompress the .zip file, and open through a web browser the "index.html" file available on the main directory. (ZIP 1823 kb). [file 1471-2105-13-42-S1.ZIP › Cloud-BioLinux-Package-Documentation/docs/gap2caf.html]

Bio-Linux Software Documentation Pages

Back to search form

## gap2caf

|  |  |
| --- | --- |
| Name | gap2caf |
| Description | **gap2caf** converts gap4 formatted databases to the common assembly format (caf).  To get a full list of command line options for gap2caf, just type: `gap2caf -h`  To do a simple conversion from gap4 format database called mydb.0, say, you could type a command like: `gap2caf_static -project mydb -version 0 -ace mydb.out` |
| Homepage | ftp://ftp.sanger.ac.uk/pub/jkb/ |
| Remote Documentation | http://www.sanger.ac.uk/Software/formats/CAF/userguide.shtml |
